# Serum YKL-40 and colorectal cancer

**DOI:** 10.1038/sj.bjc.6690238

**Published:** 1999-03

**Authors:** C Cintin, J S Johansen, I J Christensen, P A Price, S Sørensen, H J Nielsen

**Affiliations:** Department of Surgical Gastroenterology, University of Copenhagen, Hvidovre Hospital, Copenhagen, Denmark; Department of Rheumatology, University of Copenhagen, Hvidovre Hospital, Copenhagen, Denmark; The Finsen Laboratory, University of Copenhagen, Rigshospitalet, Copenhagen, Denmark; Department of Biology, University of California San Diego, La Jolla, California, USA; Department of Clinical Biochemistry, University of Copenhagen, Hvidovre Hospital, Copenhagen, Denmark

**Keywords:** carcinoembryonic antigen, colorectal cancer, metastasis, tumour invasiveness, YKL-40/HC gp-39

## Abstract

YKL-40 is a mammalian member of the chitinase protein family. Although the function of YKL-40 is unknown, the pattern of its expression suggests a function in remodelling or degradation of extracellular matrix. High serum YKL-40 has been found in patients with recurrent breast cancer and has been related to short survival. In the present study we analysed YKL-40 in preoperative sera from patients with colorectal cancer and evaluated its relation to survival. Serum YKL-40 was determined by RIA in 603 patients. Survival after operation was registered, and median follow-up time was 61 months. Three hundred and forty patients died. Sixteen per cent of the patients with Dukes' A, 26% with Dukes' B, 19% with Dukes' C and 39% with Dukes' D had high serum YKL-40 levels (adjusted for age). Analysis of serum YKL-40 as a continuous variable showed an association between increased serum YKL-40 and short survival (*P* < 0.0001). Patients with high preoperative serum YKL-40 concentration had significantly shorter survival than patients with normal YKL-40 (HR = 1.7; 95% CI: 1.3–2.1, *P* < 0.0001). Multivariate Cox analysis including serum YKL-40, serum CEA, Dukes' stage, age and gender showed that high YKL-40 was an independent prognostic variable for short survival (HR = 1.4; 95% CI: 1.1–1.8, *P* = 0.007). These results suggest that YKL-40 may play an important role in tumour invasion. © 1999 Cancer Research Campaign


*British Journal of Cancer* (1999) **79**(9/10), 1494–1499
© 1999 Cancer Research Campaign
Article no. bjoc.1998.0238

### Serum YKL-40 and colorectal cancer

**C Cintin[1], JS Johansen[2], IJ Christensen[3], PA Price[4], S Sørensen[5] and HJ Nielsen[1]**

[1]Department of Surgical Gastroenterology and [2]Department of Rheumatology, Hvidovre Hospital, University of Copenhagen, Denmark; [3]The Finsen Laboratory, Rigshospitalet, University of Copenhagen, Denmark; [4]Department of Biology, University of California San Diego, La Jolla, California, USA; and [5]Department of Clinical Biochemistry, Hvidovre Hospital, University of Copenhagen, Denmark; and the RANX05 Colorectal Cancer Study Group (Appendix)

**Summary** YKL-40 is a mammalian member of the chitinase protein family. Although the function of YKL-40 is unknown, the pattern of its expression suggests a function in remodelling or degradation of extracellular matrix. High serum YKL-40 has been found in patients with recurrent breast cancer and has been related to short survival. In the present study we analysed YKL-40 in preoperative sera from patients with colorectal cancer and evaluated its relation to survival. Serum YKL-40 was determined by RIA in 603 patients. Survival after operation was registered, and median follow-up time was 61 months. Three hundred and forty patients died. Sixteen per cent of the patients with Dukes' A, 26% with Dukes' B, 19% with Dukes' C and 39% with Dukes' D had high serum YKL-40 levels (adjusted for age). Analysis of serum YKL-40 as a continuous variable showed an association between increased serum YKL-40 and short survival ($P < 0.0001$). Patients with high preoperative serum YKL-40 concentration had significantly shorter survival than patients with normal YKL-40 (HR = 1.7; 95% CI: 1.3–2.1, $P < 0.0001$). Multivariate Cox analysis including serum YKL-40, serum CEA, Dukes' stage, age and gender showed that high YKL-40 was an independent prognostic variable for short survival (HR = 1.4; 95% CI: 1.1–1.8, $P = 0.007$). These results suggest that YKL-40 may play an important role in tumour invasion.

**Keywords:** carcinoembryonic antigen; colorectal cancer; metastasis; tumour invasiveness; YKL-40/HC gp-39

Patients with advanced colorectal carcinoma have a poor prognosis. Although approximately 70% of the patients with primary disease may undergo an apparently curative resection, 40% will develop recurrent disease within 5 years (McArdle et al, 1990). Liver metastases are the major determinant of reduced survival (Finley and McArdle, 1983) but it is still difficult to predict patients at risk. Follow-up regimens after resection for primary colorectal cancer in general consist of periodic interval history, physical examinations and endoscopic surveillance. The usefulness of analysing consecutive serum carcinoembryonic antigen (CEA) levels has been questioned (Kievit and Van der Velde, 1990; Virgo et al, 1995) but CEA is still used as an eventual predictor of residual disease or metastases (Lucha et al, 1997).

YKL-40* is a mammalian member of the chitinase protein family which has no chitinase activity (Nyirkos et al, 1990; Hakala et al, 1993; Johansen et al, 1993; Shackelton et al, 1995; Hu et al, 1996; Kirkpatrick et al, 1997; Rehli et al, 1997; Renkema et al, 1998) but does bind chitin (Renkema et al, 1998). Although the physiological function of YKL-40 is unknown, the pattern of its expression in normal and disease state suggests a function in remodelling or degradation of extracellular matrix. YKL-40 is secreted in large amounts in vitro by the MG63 human osteosarcoma cell line (Johansen et al, 1992) and is expressed selectively by murine mammary tumours initiated by *neu*/*ras* oncogenes but not by c-*myc* or *int*-2 oncogenes (Morrison and Leder, 1994). Furthermore, YKL-40 is synthesized by activated macrophages (Krause et al, 1996; Kirkpatrick et al, 1997; Renkema et al, 1998) and the protein is present in the specific granules of neutrophils and is exocytosed by activation (Volck et al, 1998).

The protein can be determined in serum by radioimmunoassay (RIA) (Johansen et al, 1993). Recently, we demonstrated that serum YKL-40 levels are elevated in patients with recurrent breast cancer and metastases to bone or viscera and may be used as a prognostic biochemical marker of survival (Johansen et al, 1995). We have therefore studied whether YKL-40 is elevated in patients with primary colorectal cancer and whether YKL-40 can be used as a prognostic marker of survival.

### MATERIALS AND METHODS

### Patients

The study included 603 patients, 355 males and 248 females, with a median age of 69 years (range 33–91 years), who underwent primary elective large bowel resection for colorectal cancer. Three hundred and fifty-five patients had cancer of the colon and 250 patients had rectal cancer. The patients, described in details elsewhere (Nielsen et al, 1998), participated in a national multicentre study, comprising 20 Danish hospital centres, performed between 1990 and 1997. Patients estimated as having a shorter survival than 3 months were not included. Dukes' stage and survival after the operation were registered. None of the patients had an infection or was treated with steroids at time of operation. None of the patients received postoperative adjuvant chemotherapy. Median follow-up time was 61 months (range 45–75 months). The patients were followed for death/survival by using their health security

*Received 20 May 1998*
*Revised 30 September 1998*
*Accepted 14 October 1998*

*Correspondence to:* HJ Nielsen

*YKL-40 has been named after its molecular weight (40 kDa) and the one letter code for its three N-terminal amino acids (Johansen et al, 1992). The protein is also called human cartilage glycoprotein-39 (HC gp-39) (Hakala et al, 1993).

number (CPR) in the central national registry. No patients were lost at follow-up. The endpoint was death of all causes and 340 patients died. Twenty patients who died within 1 month from surgery of causes other than cancer were censored. The study was performed in agreement with the Helsinki II declaration. The research protocol was approved by the local ethical committee. The patients were informed about the study verbally and in writing. All gave their written consent. The patients were informed about the possibility of withdrawing from the study at any time.

### Controls

The controls comprised 260 persons, 116 males and 144 females, with a median age of 48 years (range 18–79 years) (Johansen et al, 1996). The controls were blood donors who attended the Regional Blood Transfusion Services at Hvidovre Hospital, people working at different museums in Copenhagen and people living in a shared house for elderly. All were healthy, were not taking any medicine, and had no signs or clinical symptoms of cancer, joint, liver, metabolic or hormonal disease. The median serum YKL-40 level was 102 µg $l^{-1}$ (range 38–514 µg $l^{-1}$, upper 95th percentile = 247 µg $l^{-1}$), with a weak correlation to age (Spearman = 0.30). There was no difference between gender ($P$ = 0.65, Wilcoxon 2-sample test). A normal reference region was calculated on the log transformed YKL-40 values as described by Royston (1991) adjusting for age, the upper 95th per cent confidence limit was chosen for the limit.

### Biochemical analysis

Blood samples were taken in the morning before surgery and serum was separated from cellular elements by centrifugation within 1 h of sampling. All serum samples were stored at – 80°C until analysis. Serum YKL-40 was determined by RIA (Johansen et al, 1993) using rabbit antibody raised against human YKL-40. Purified human YKL-40 was used for standard and tracer. The intra-assay and inter-assay variations were < 6.5% and < 12% respectively, and the sensitivity was 20 µg $l^{-1}$. CEA was measured in serum using the Immulite CEA assay (Euro/DPC Ltd, Llanberis, Gwynedd, UK).

### Statistical analysis

The statistical analysis was done with SAS$^R$ (SAS Institute, Cary, NC, USA). Tests for homogeneity between covariates were done using the chi-square. Survival curves were estimated by the Kaplan–Meier method. The log-rank test was used for test of homogeneity between strata. Multivariate survival analyses were performed with the Cox proportional hazards model. The assumption of proportional hazards was verified graphically. The endpoint was death of all causes (overall survival). The serum YKL-40 covariate was dichotomized by the normal reference region as described above. The other covariates included in the multivariate analysis of Cox were serum CEA [dichotomized by its median level (3.8 µg $l^{-1}$)], Dukes' stage (entered as indicator variable), gender and age.

### RESULTS

The distribution by Dukes' staging was 58 in A, 223 in B, 175 in C and 147 in D. Median follow-up time was 61 months (range 45–75 months) and in this period 340 patients died. The median preoperative serum YKL-40 concentration in all patients was 180 µg $l^{-1}$ (range 56–2709 µg $l^{-1}$). The number of patients with YKL-40 levels above the age-corrected 95th percentile of normal controls was 159. There was no significant difference between high serum YKL-40 and gender ($P$ = 0.07, Wilcoxon rank sum test) but there was a relatively weak correlation to age (Spearman = 0.30, $P$ < 0.0001). Sixteen per cent of the patients with Dukes' A, 26% with Dukes' B, 19% with Dukes' C, and 39% with Dukes' D had increased levels of serum YKL-40, i.e. a serum YKL-40 level above the age-corrected 95th percentile of normal controls. Figure 1 shows the individual serum YKL-40 values according to Dukes stage. The chi-square showed a significant association between serum YKL-40 and Dukes' stage ($P$ = 0.001).

Analysis of the serum YKL-40 value as a continuous variable showed a highly significant association between increased serum YKL-40 and short survival ($P$ < 0.0001). Figure 2 illustrates the survival plot when the patients were grouped by quartiles according to their preoperative serum YKL-40 level: Group 1, patients with a serum YKL-40 ≤ 120 µg $l^{-1}$ ($n$ = 161); Group 2, patients with a serum YKL-40 > 120 and ≤ 180 µg $l^{-1}$ ($n$ = 141); Group 3, patients with a serum YKL-40 > 180 and ≤ 304 µg $l^{-1}$ ($n$ = 152) and Group 4, patients with a serum YKL-40 > 304 µg $l^{-1}$ ($n$ = 149).

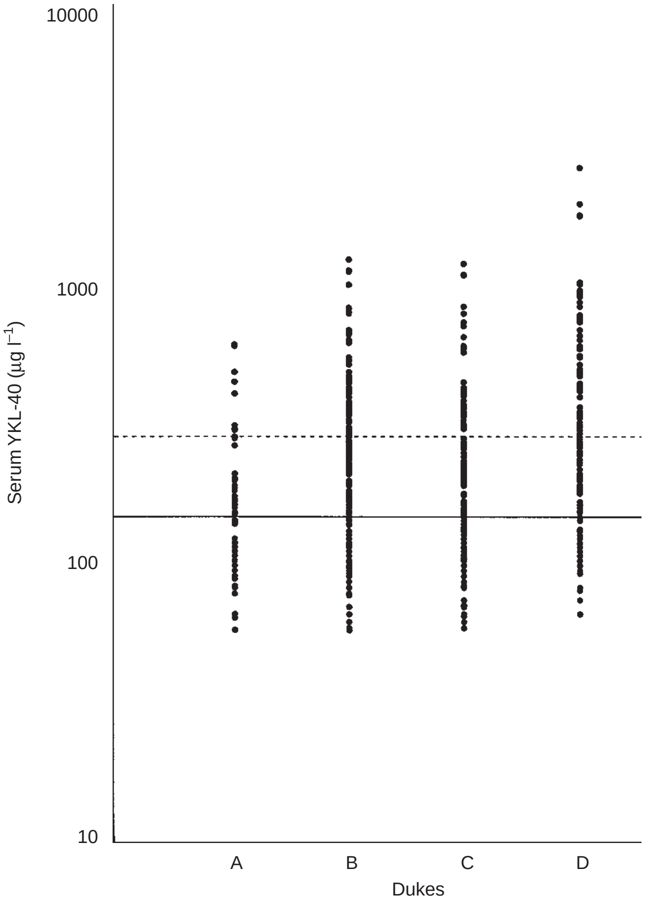

**Figure 1** The figure shows the YKL-40 values in each Dukes stage. The solid and dotted lines indicate the mean and upper 95% limit values for YKL-40 predicted for normal 70-year-old subjects (the median age for colon cancer subjects in the present study is 69 years)

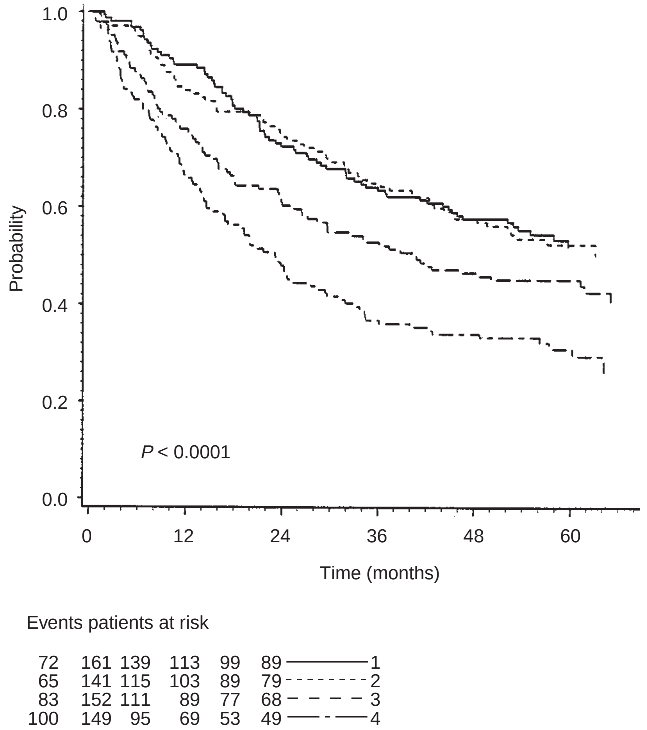

**Figure 2** The impact of serum YKL-40 level on overall survival of colorectal cancer patients. Patients were divided into four groups according to the serum level of YKL-40 obtained preoperatively: Group 1 (—), patients with serum YKL-40 ≤ 120 µg l⁻¹ (*n* = 161); Group 2 (- -), patients with a serum YKL-40 > 120 and ≤ 180 µg l⁻¹ (*n* = 141); Group 3 (–), patients with a serum YKL-40 > 180 and ≥ 304 µg l⁻¹ (*n* = 152); and Group 4 (– -), patients with a serum YKL-40 > 304 µg l⁻¹ (*n* = 149). The number of events are shown for each group at the left, and the number of patients at risk are shown for 0, 12, 24, 36 and 48 months

**Table 1** Independent prognostic variables from the Cox multivariate analysis

| Covariate | *P*-value | HR | 95% CI |
|---|---|---|---|
| YKL-40 (high vs normal) | 0.007 | 1.4 | 1.1–1.8 |
| CEA (high vs. low) | 0.10 | | |
| Age (years) | 0.0002 | 1.4[a] | 1.3–1.5 |
| Dukes' B | 0.003 | 3.2 | 1.5–6.9 |
| (vs Dukes A) | | | |
| Dukes' C | 0.0001 | 7.0 | 3.3–15.1 |
| (vs Dukes A) | | | |
| Dukes' D | 0.0001 | 27.2 | 12.6–58.4 |
| (vs Dukes A) | | | |
| Gender (male/female) | 0.1 | | |

HR, relative hazard ratio; CI, confidence interval. [a]HR for two patients differing in age by 15 years.

When all the patients were grouped by a high (versus normal, age-corrected) preoperative serum YKL-40 concentration, the group with high YKL-40 had significantly shorter survival than patients with a normal preoperative serum YKL-40 [Hazard ratio (HR) of 1.7; 95% confidence interval (CI): 1.3–2.1, *P* < 0.0001]. The Kaplan–Meier plot for all patients is shown in Figure 3A, Dukes' B patients in Figure 3B, Dukes' C patients in Figure 3C and Dukes' D patients in Figure 3D. The Kaplan–Meier plot was not evaluated for patients with Dukes' A due to the low number of patients and events.

Univariate survival analysis of the other included covariates showed that Dukes' stage was highly significant (*P* < 0.0001), as well as age (in years, *P* = 0.002) and CEA (*P* < 0.0001, HR = 1.9, 95% CI: 1.5–2.3), whereas gender was not (*P* = 0.17).

A multivariate Cox analysis including serum YKL-40, serum CEA, Dukes' stages, age and gender showed that a high YKL-40 was an independent prognostic parameter for short survival, with a HR of 1.4 (95% CI: 1.1–1.8, *P* = 0.007) (Table 1). Dukes' staging was the strongest independent prognostic variable and age was also a significant independent prognostic parameter of survival. Serum CEA and gender were not significant prognostic parameters of survival.

A multivariate Cox analysis including only serum YKL-40 and serum CEA (*n* = 598) showed that both parameters were independent prognostic parameters of survival (*P* < 0.0001) with a HR of 1.6 (95% CI: 1.3–2.0) for serum YKL-40 and 1.9 (95% CI: 1.5–2.3) for serum CEA. There was no significant difference between high YKL-40 plus low CEA compared to low YKL-40 plus high CEA (*P* = 0.15). When YKL-40 and CEA were combined, a highly significant separation (*P* < 0.0001) was obtained between patients with both high serum YKL-40 and high serum CEA (*n* = 84) and patients with normal levels of both parameters (*n* = 231) with a HR of 3.3 (95% CI: 2.4–4.4) (Figure 4).

### DISCUSSION

Dukes' staging is a well-established strong prognostic indicator of survival in patients suffering from colorectal cancer (Dukes and Bussey, 1958; Wiggers et al, 1988; Ståhle et al, 1989). However, a considerable variation in prognosis has been demonstrated within each stage (Jass et al, 1987; Newland et al, 1987) and some patients with Dukes' stage B have a poorer prognosis than patients in Dukes' C. Several studies have been performed to find new biochemical markers in order to identify patients at high risk for recurrence, who might be candidates for additional therapy after surgery. There are numerous reports on CEA in screening and follow-up of patients with colorectal cancer, but this marker seems to be of limited clinical use (Kievit and Van der Velde, 1990; Virgo et al, 1995; Lucha et al, 1997). Nevertheless, the most frequently used marker is still CEA.

We recently examined serum YKL-40 in patients with cancer mamma and found that high levels of YKL-40 are a prognostic indicator of a significantly shorter survival (Johansen et al, 1995). In the present study we evaluated the possible relationship between the preoperative level of YKL-40 in serum and the survival of the patients after surgery for colorectal cancer. A strong association was found between short survival and high preoperative YKL-40 levels. A significant relation was also found between serum YKL-40 and Dukes' stage, but multivariate Cox analysis showed that serum YKL-40 was a prognostic variable of survival,

 *© Cancer Research Campaign 1999*

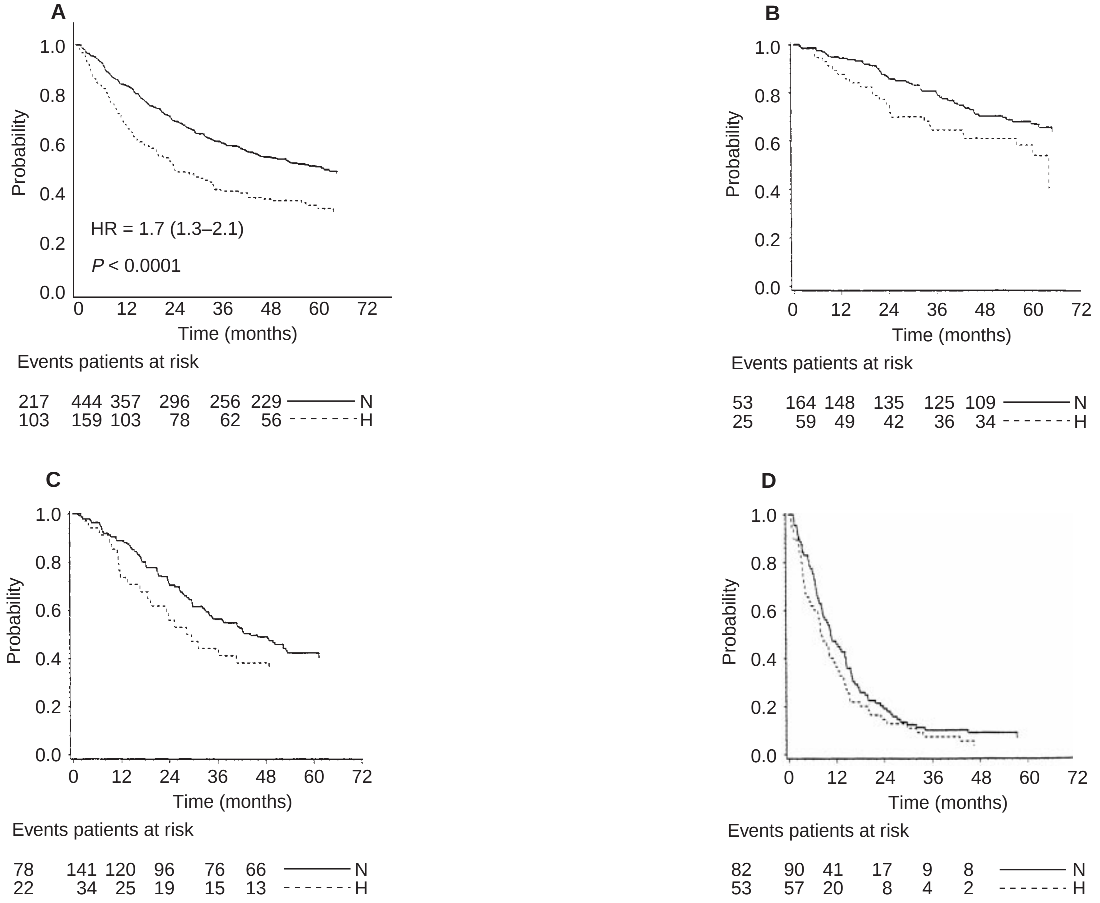

**Figure 3** The impact of serum YKL-40 level on overall survival of colorectal cancer patients. Patients were grouped by a high (vs normal) preoperative serum YKL-40 concentration adjusted for age. The cut-off limit used was 95th confidence limit of healthy age-matched subjects: patients with normal serum YKL-40 (N —) and patients with elevated serum YKL-40 levels (H- - -). **A** illustrates the results of all patients (HR = 1.7, 95%CI: 1.3–2.1, $P = 0.0001$). **B** illustrates the results in patients with Dukes' B (HR = 1.6, 95% CI: 1.0–2.5 $P = 0.07$), **C**, the patients with Dukes' C (HR = 1.4, 95% CI: 0.8–2.2, $P = 0.21$) and D the results in patients with Dukes' D (HR = 1.3, 95% CI: 0.9–1.8, $P = 0.15$). The number of events are shown for each group at the left, and the number of patients at risk are shown for 0, 12, 24, 36 and 48 months independent of Dukes' stage. If preoperative high levels of YKL-40 do prove to identify patients in Dukes' B and C with a high risk of recurrence, more intensive follow-up and treatment could be given to these patients, such as adjuvant therapy or reoperation.

The precise sources of YKL-40 which lead to elevated serum levels of the protein in some colorectal cancer patients are not yet known. Serum YKL-40 could in principle arise from secretion by the tumour cells themselves, from secretion by inflammatory cells, and from secretion by normal cells in areas of the colon adjacent to the tumour. We are currently investigating the expression of YKL-40 in colon cancer biopsies using immunohistochemical methods. Preliminary data show that some colon cancers stain intensely for YKL-40 while other colon cancers are completely negative. Normal intestinal epithelium distant from the areas of neoplasia are negative. Although some YKL-40 staining can be seen in mononuclear cells located in the connective tissue, there is no difference in mononuclear staining between connective tissue areas adjacent to the tumour and areas distant from the tumour (personal observation). Some support for the hypothesis that YKL-40 may be secreted by a subset of colorectal tumours is provided by the observation that the protein is strongly expressed by murine mammary tumours initiated by *neu*/*ras* oncogenes but is not expressed by mammary tumours initiated by c-*myc* or by *int*-2 (Morrison and Leder, 1994). It is of interest to note that the investigators who made these observations on mammary tumours independently concluded that YKL-40 could be a marker for a subset of human breast cancers, without knowledge of our contemporaneous study that showed that serum YKL-40 is, in fact, a prognostic indicator of survival in patients with recurrent breast cancer (Johansen et al, 1995).

If elevated levels of serum YKL-40 do primarily reflect secretion from a subset of colorectal tumours, then the poor prognosis of patients with elevated serum YKL-40 suggests that YKL-40 expression may be associated with the ability of a tumour cell to invade normal tissues and to metastasize to distant sites. It is also possible that the as yet unknown function of YKL-40 itself may be important to an aspect of tumour invasiveness.

Recent studies have shown that YKL-40 is a chitin-binding lectin (Renkema et al, 1998). Chitin is a homopolymer of N-acetyl-D-glucosamine in β 1–4 linkage, which is found in the cell wall of fungi and in the exoskeleton of insects, crustaceans and arthropods, but not in any mammalian tissue (Skjak-Braek et al, 1989). The functional ligand for the chitin binding site in YKL-40 is not presently known. Other lectins have been found at elevated

© Cancer Research Campaign 1999

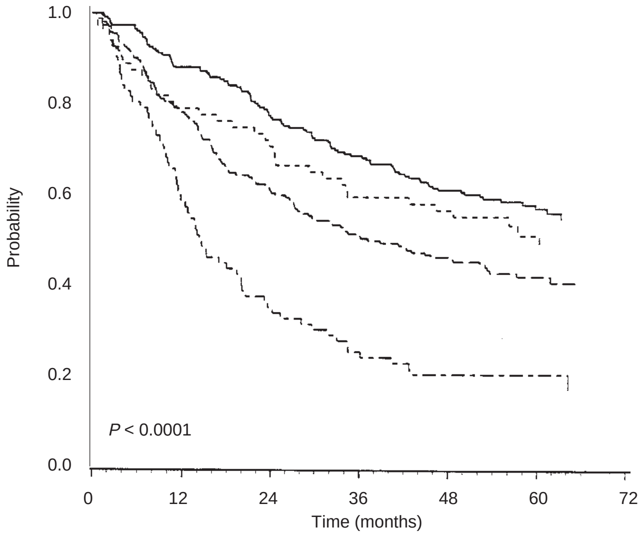

Events patients at risk

| | | | | | | |
|---|---|---|---|---|---|---|
| 97 | 231 | 198 | 173 | 154 | 137 | ——— 1 |
| 35 | 73 | 56 | 50 | 42 | 40 | - - - - - - - 2 |
| 117 | 209 | 156 | 121 | 101 | 91 | — — — - 3 |
| 66 | 84 | 47 | 28 | 20 | 16 | —— - — 4 |

**Figure 4** The impact of combinations of serum YKL-40 and serum CEA levels on overall survival of colorectal cancer patients. The patients were divided into four groups according to the serum level of YKL-40 and CEA obtained at time of operation. Patients were grouped by a high (vs normal) preoperative serum YKL-40 concentration adjusted for age. The cut-off limit used was 95th confidence limit of healthy age-matched subjects. Serum CEA concentration was dichotomized by its median level (3.8 $\mu g\ l^{-1}$). Group 1 (—) patients with normal levels of both markers; Group 2 (- -), patients with high YKL-40 but normal CEA; Group 3 (–), patients with normal YKL-40 but high CEA; and Group 4 (– -), patients with high levels of both markers. The number of events are shown for each group at the left, and the number of patients at risk are shown for 0, 12, 24, 36 and 48 months. For low CEA, there is no significant difference between low and high YKL-40, whereas for high CEA there is a significant difference between low and high YKL-40 ($P < 0.0001$, HR = 2.0 (1.5–2.7)

levels in a variety of neoplastic cells, and studies suggest that some of these lectins are involved as adhesion molecules for tumour metastasis *in vivo* (Raz et al, 1990; Schoeppner et al, 1995; Bressalier et al, 1996). In colon cancer lectin binding has been linked to tumour progression and K-*ras* activation (Wojciechowicz et al, 1995).

As part of this multicentre colorectal cancer study, each primary tumour has been fixed for possible histological analyses and serum samples have been obtained periodically following surgery for removal of the primary tumour. In future studies we intend to use immunohistochemistry to establish the pattern of YKL-40 expression in colorectal tumours obtained from patients with elevated as well as normal serum YKL-40 levels, and we will examine the longitudinal relationship between serum YKL-40 levels and survival.

### ACKNOWLEDGEMENTS

This study was supported by grants from: Dagmar Marshalls Foundation, Overlæge Johan Boserup og Lise Boserups Legat, Michaelsen Fonden, Glaxo Research Ltd, UK, The Ingeborg Roikjer Foundation, Direktør Jacob Madsen og Hustru Olga Madsens Fond, The Krista & Viggo Petersen Foundation and Apotekerfonden of 1991.

The expert technical assistance of Kirsten Vangsgaard, Department of Surgical Gastroenterology, Hvidovre Hospital and Susanne Munch, Department of Rheumatology, Hvidovre Hospital is greatly appreciated.

### REFERENCES

Bresalier RS, Byrd JC, Wang L and Raz A (1996) Colon cancer mucin: a new ligand for the β-galactoside-binding protein galectin-3. *Cancer Res* **56**: 4354–4357

Dukes C and Bussey HJR (1958) The spread of rectal cancer and its effect on prognosis. *Br J Cancer* **12**: 309–320

Finlay I and McArdle CS (1983) Effect of occult hepatic metastases on survival after curative resection for colorectal carcinoma. *Gastroenterology* **85**: 596–599

Hakala BE, White C and Recklies AD (1993) Human cartilage gp-39, a major secretory product of articular chondrocytes and synovial cells, is a mammalian member of a chitinase protein family. *J Biol Chem* **268**: 5803–5810

Hu B, Trinh K, Figueira WF and Price PA (1996) Isolation and sequence of a novel human chondrocyte protein related to mammalian members of the chitinase protein family. *J Biol Chem* **271**: 19415–19420

Jass JR, Love SB and Northover JM (1987) A new prognostic classification of rectal cancer. *Lancet* **1**: 1303–1306

Johansen JS, Williamson MK, Rice JS and Price PA (1992) Identification of proteins secreted by human osteoblastic cells in culture. *J Bone Miner Res* **7**: 501–512

Johansen JS, Jensen HS and Price PA (1993) A new biochemical marker for joint injury. Analysis of YKL-40 in serum and synovial fluid. *Br J Rheumatol* **32**: 949–955

Johansen JS, Cintin C, Jørgensen M, Kamby C and Price PA (1995) Serum YKL-40: a new potential marker of prognosis and location of metastases of patients with recurrent breast cancer. *Eur J Cancer* **31A**: 1437–1442

Johansen JS, Hvolris J, Hansen M, Backer V, Lorenzen I and Price PA (1996) Serum YKL-40 levels in healthy children and adults. Comparison with serum and synovial fluid levels of YKL-40 in patients with osteoarthritis or trauma of the knee joint. *Br J Rheumatol* **35**: 553–559

Kievit J and Van de Velde CJH (1990) Utility and cost of carcinoembryonic antigen monitoring in colon cancer follow-up evaluation. *Cancer* **65**: 2580–2587

Kirkpatrick RB, Emery JG, Connor JR, Dodds R, Lysko PG and Rosenberg M (1997) Induction and expression of human cartilage glycoprotein 39 in rheumatoid inflammatory and peripheral blood monocyte-derived macrophages. *Exp Cell Res* **237**: 46–54

Krause SW, Rehli M, Kreutz M, Schwarzfischer L, Paulauskis JD and Andreesen R (1996) Differential screening identifies genetic markers of monocyte to macrophage maturation. *J Leukoc Biol* **60**: 540–545

Lucha PA, Rosen L, Olenwine JA, Reed JF, Riether RD, Stasik JJ and Khubchandani IT (1997) Value of carcinoembryonic antigen monitoring in curative surgery for recurrent colorectal carcinoma. *Dis Colon Rectum* **40**: 145–149

McArdle CS, Hole D, Hansell D, Blumgart LH and Wood CB (1990) Prospective study of colorectal cancer in the West of Scotland: 10 year follow-up. *Br J Surg* **77**: 280–282

Morrison BW and Leder P (1994) *neu* and *ras* initiate murine mammary tumors that share genetic markers generally absent in *c-myc* and *int*-2-initiated tumors. *Oncogene* **9**: 3417–3426

Newland RC, Chapuis PH and Smyth EJ (1987) The prognostic value of substaging colorectal carcinoma. A prospective study of 1117 cases with standardized pathology. *Cancer* **60**: 852–857

Nielsen HJ, McArdle CS, Moesgaard F and The RANX05 Study Group (1998) The effect of ranitidine on long-term survival in primary colorectal cancer. *G I Cancer* **2**: 227–233

Nyirkos P and Golds EE (1990) Human synovial cells secrete a 39 kDa protein similar to a bovine mammary protein expressed during the non-lactating period. *Biochem J* **268**: 265–268

Raz A, Zhu D, Hogan V, Shah N, Raz T, Karkash R, Pazerine G and Carmi P (1990) Evidence for the role of 34-kDa galactoside-binding lectin in transformation and metastasis. *Int J Cancer* **46**: 871–877

Rehli M, Krause SW and Andreesen R (1997) Molecular characterization of the gene for human cartilage gp-39 (CHI3L1), a member of the chitinase protein family and marker for late stages of macrophage differentiation. *Genomics* **43**: 221–225

Renkema GH, Boot GR, Au FL, Donker-Koopman WE, Strijland A, Muijsers AO, Hrebicek M and Aerts JMFG (1998) Chitotriosidase, a chitinase, and the 39-

*© Cancer Research Campaign 1999*

kDa human cartilage glycoprotein, a chitin-binding lectin, are homologues of family 18 glycosyl hydrolases secreted by human macrophages. *Eur J Biochem* **251**: 504–509

Royston P (1991) Constructing time-specific reference ranges. *Statist Med* **10**: 675–690

Shoeppner HL, Raz A, Ho SB and Bresalier RS (1995) Expression of an endogenous galactose-binding lectin correlates with neoplastic progression in the colon. *Cancer* **75**: 2818–2826

Shackelton LM, Mann DM and Millis AJT (1995) Identification of a 38-kDa heparin binding glycoprotein (gp38k) in differentiating vascular smooth muscle cells as a member of a group of proteins associated with tissue remodeling. *J Biol Chem* **270**: 13076–13083

Skjak-Braek G, Anthonsen T and Sandford PA (1989) *Chitin and Chitosan*: *Sources, Chemistry, Biochemistry, Physical Properties and Applications*. Elsevier Applied Science: New York

Ståhle E, Glimelius B, Bergstrom R and Pahlman L (1989) Preoperative prediction of outcome in patients with rectal and rectosigmoid cancer. *Cancer* **63**: 1831–1837

Virgo KS, Vernava AM, Longo WE, McKirgan LW and Johnson FE (1995) Cost of patient follow-up after potentially curative colorectal cancer treatment. *JAMA* **23**: 1837–1841

Volck B, Price PA, Johansen JS, Sørensen O, Benfield T, Nielsen HJ, Calafat J and Borregaard N (1998) YKL-40, a mammalian member of the bacterial chitinase family, is a matrix protein of specific granules in human neutrophils. *Proc Assoc Am Phys* **110**: 351–360

Wiggers T, Arends JW, Schutte B and Volovics Bosman FT (1988) A multivariate analysis of pathological prognostic indicators in large bowel cancer. *Cancer* **61**: 386–395

Wojciechowitcz DC, Park PY and Baty PB (1995) β1–6 branching of N-linked carbohydrate is associated with K-ras mutation in human colon carcinoma cell lines. *Biochem Biophys Res Commun* **212**: 758–766

### APPENDIX

The following investigators participated in The RANX05 Colorectal Cancer Study Group.

| | |
|---|---|
| Bispebjerg Hospital: | S Schulze, MD, DMSc |
| | J Thorup, MD |
| | P Wille-Jørgensen, MD, DMSc |
| Bornholm Hospital: | E Bentzen, MD |
| Frederiksberg Hospital: | I Christoffersen, MD |
| | P Møller, MD |
| Frederikssund Hospital: | L Banke, MD, DMSc |
| | D Froberg, MD |
| Gentofte Hospital: | FW Henriksen, MD, DMSc |
| | P Crone, MD |
| Glostrup Hospital: | P Hesselfeldt, MD |
| | B Hempel Sparsø, MD |
| | K Lindorff Larsen, MD |
| Helsingør Hospital: | T Asmussen, MD |
| | J Heiner, MD |
| Hillerød Hospital: | O Hart Hansen, MD, DMSc |
| | H Flyger, MD, PhD |
| | P Jess, MD, DMSc |
| Holbæk Hospital: | J Iversen, MD |
| | J La Cour-Andersen, MD, DMSc |
| | B Vennits, MD |
| Hvidovre Hospital: | F Moesgaard, MD, DMSc |
| | J Hammer, MD |
| Hørsholm Hospital: | A Fischer, MD, DMSc |
| | H Galatius, MD |
| Kalundborg Hospital: | L Naver, MD |
| Køge Hospital: | D Teilum, MD |
| Nykøbing Falster Hospital: | L Holbraad, MD |
| Næstved Hospital: | O Iversen, MD, DMSc |
| | J Nymark, MD |
| | O Roikjær, MD |
| Rigshospitalet: | LB Svendsen, MD, DMSc |
| | L Vedel, MD |
| Roskilde Hospital: | L Palm, MD, DMSC |
| | KC Rasmussen, MD |
| Slagelse Hospital: | J Friis, MD |
| | C Lanng, MD |
| | K Wiboltt, MD |
| Stege Hospital: | NC Jensen, MD |
| | N Hoffman, MD |
| Sundby Hospital: | T Larsen, MD |
| | J Packler, MD |

© Cancer Research Campaign 1999
